# 3D Printing of Textured Soft Hybrid Meat Analogues

**DOI:** 10.3390/foods11030478

**Published:** 2022-02-06

**Authors:** Tianxiao Wang, Lovedeep Kaur, Yasufumi Furuhata, Hiroaki Aoyama, Jaspreet Singh

**Affiliations:** 1 School of Food and Advanced Technology, Massey University, Palmerston North 4442, New Zealand; wtx0221@gmail.com; 2 Riddet Institute, Palmerston North 4442, New Zealand; 3 Ajinomoto Co., Inc., Suzuki-cho 3-1, Kawasaki-ku, Kawasaki-shi 210-0801, Japan; yasufumi.furuhata.pd7@asv.ajinomoto.com (Y.F.); hiroaki_aoyama@ajinomoto.com (H.A.)

**Keywords:** food 3D printing, hybrid meat analogues, rheological properties, pea protein isolate, chicken

## Abstract

Meat analogue is a food product mainly made of plant proteins. It is considered to be a sustainable food and has gained a lot of interest in recent years. Hybrid meat is a next generation meat analogue prepared by the co-processing of both plant and animal protein ingredients at different ratios and is considered to be nutritionally superior to the currently available plant-only meat analogues. Three-dimensional (3D) printing technology is becoming increasingly popular in food processing. Three-dimensional food printing involves the modification of food structures, which leads to the creation of soft food. Currently, there is no available research on 3D printing of meat analogues. This study was carried out to create plant and animal protein-based formulations for 3D printing of hybrid meat analogues with soft textures. Pea protein isolate (PPI) and chicken mince were selected as the main plant protein and meat sources, respectively, for 3D printing tests. Then, rheology and forward extrusion tests were carried out on these selected samples to obtain a basic understanding of their potential printability. Afterwards, extrusion-based 3D printing was conducted to print a 3D chicken nugget shape. The addition of 20% chicken mince paste to PPI based paste achieved better printability and fibre structure.

## 1. Introduction

Meat-based products are popular among consumers due to their unique taste, texture and nutritional values [1]. With the development of technology, meat is becoming more accessible to humans’ daily diets. According to predictions, the demand of meat will continue to grow in the future [2]. However, growing meat consumption is associated with increasing environmental concerns, including large land use and green gas emission [3,4,5]. To ensure sustainability, alternative diets or food sources have been suggested to decrease the average individual meat consumption [6]. Meat analogue is a type of food considered as a replacer that mimics characteristics of meat.

Traditional meat analogues have been created and developed for many centuries. These meat analogues were generally made by vegetables or plants rich in protein, to deal with low meat accessibility or due to religious reasons in different parts of the world [7,8,9]. Due to the limitations of the traditional processing technique, traditional meat analogues could not properly simulate the sensory characteristics and texture of meat. Therefore, scientists have started researching new methods to improve the quality of meat analogues [9,10].

Currently, the most common technology producing meat analogue is high-moisture extrusion. It can be used on various plant protein sources and produce different kinds of meat analogues [9,11,12]. The percentages of moisture in high-moisture extrusion normally vary from 40 to 80% [13]. The extruded products tend to show higher similarity to meat, compared with conventional meat analogues. Other novel technologies such as shearing, and spinning have also been developed to further imitate the meat-like fibres and microstructure [14,15,16]. However, these methods have not been widely promoted on an industrial scale. Aside from its meat-like structure, the nutritional value and other physical sensations of meat analogue also need to be improved.

Consumers’ preference plays an important role in the commercialisation of meat analogues. Currently, the challenges of developing meat analogues mainly include lower nutrition quality of plant proteins, lack of meaty sensations and high price [17,18]. To improve the nutritional and sensorial characteristics of meat analogues, one option is to add low value animal proteins to formulate a hybrid meat analogue. Animal proteins are generally known to be nutritionally superior to plant proteins.

Additionally, it is worthwhile to develop some meat analogues, which are suitable for the elderly. There are increasing numbers of elderly that need to be fed. However, the decreasing tooth strength with aging limits the elderly’s’ food choices [19].

Three-dimensional printing is a novel technology, which could be introduced into food manufacturing to modify food structure and texture [20]. In addition, it creates desirable food shapes and improves nutrient selection [21]. Because of these, it has been used to produce some health care food products for the elderly. Aged people would be benefited because they have more options, instead of simply consuming conventional pureed food. Scientists have already shown their interest in producing meat analogues from plant proteins through 3D printing. Some printed plant-based meat products with different shapes have been reported [22,23,24]. However, there is no available literature on the evaluation of properties of printed meat analogue. The printing of hybrid meat analogue has also not yet been mentioned by currently available research.

Conventionally, soy is the most common material to produce meat analogues. However, pea has fewer food allergy issues and GMO concerns than soy [25]. The interest in research on pea protein has increased in recent years, especially for simulating chicken products [26,27]. Hence, pea protein and chicken were selected as the main materials to produce printed soft hybrid meats.

The main objectives of this study were to: (1) Develop a printable formulation mainly made from pea protein isolate and chicken mince paste. (2) Optimize the formulation to printing process based on rheology and extrusion tests.

## 2. Materials and Methods

### 2.1. Materials

Pea protein isolate (PPI) (containing 80% protein) was purchased from Davis Food Ingredients (Palmerston North, New Zealand). Commercially available chicken mince (typically 95% meat and 5% fat) was purchased from a local market (Palmerston North, New Zealand). It was then blended with a Moulinex Food blender (Masterchef 650, Groupe Seb New Zealand, Auckland, New Zealand) and finely minced by a Silverson L4RT High shear mixer (Advanced Packaging System Limited, Auckland, New Zealand) as much as possible into a paste-like texture.

Other materials included pre-gelatinized maize starch (Hi-Maize 1043; Ingredion ANZ Pty Ltd., Auckland, New Zealand), beef fat (Premium 100% pure beef dripping, Farmland foods, purchased from a local market, Palmerston North, New Zealand) and soy lecithin (Hawkins Watts Ltd., Auckland, New Zealand).

### 2.2. Sample Preparation

The formulations of PPI based pastes and PPI-chicken pastes (Table 1) were finalized based on preliminary extrusion trials. Soy lecithin was mixed and dispersed in water (69%) with the help of a MicroMix stick blender (Robot Coupe, 220W, Robot-Coupe Australia Pty Ltd., Auckland, New Zealand). Then, water containing soy lecithin, PPI (24%), maize starch (3.6%) and beef fat (2.4%) were added and mixed in a Moulinex food blender (Masterchef 650). All the ingredients were blended for 2 min to prepare a paste. The blended paste was transferred into metal beakers. The metal beaker containing the paste was placed in a pot containing boiling water and heated on a hotplate (MR 3001, Heidolph, Schwabach, Germany) for 10 min. The samples were further mixed during heating, using a Silverson L4RT high shear mixer (Advanced Packaging System Ltd., New Zealand) at 4000 rpm (556× *g*). The cooked paste was naturally cooled down to room temperature and used for further experimentation. Fresh pastes were prepared before each experiment.

In PPI-chicken pastes (PCP) samples, the amount of added starch, fat, soy lecithin and total moisture was same as the PPI paste. The total dry matter in the chicken paste was aimed to replace PPI powder by 20 and 50%. When PPI paste was prepared, it was mixed with a certain amount of raw chicken paste, as shown in Table 1, using a Moulinex food blender (Masterchef 650) for 2 min.

### 2.3. Rheological Properties

Rheological properties of PPI and PCP pastes were studied according to the methodology described by Wang et al. [28] with slight modifications, using a dynamic rheometer (AR-G2, TA Instruments, New Castle, DE, USA). Temperature and frequency sweep experiments were performed on raw and cooked pastes, respectively. Steady shear viscosity tests were performed on cooked PPI pastes. A 40 mm parallel steel plate geometry was chosen to test of all samples, and a gap of 2 mm was set between two plates. The strain was set as 0.4%, which ensured all samples were in their linear viscoelastic region. The measurements on each selected sample were conducted in triplicate. Data were collected and analysed by TA software (TA Universal Analysis Version 4.5A, TA Instruments, New Castle, DE, USA).

#### 2.3.1. Temperature Sweeps

A temperature sweep test aims to find how the viscoelastic properties of experimental samples change with heating and cooling. Four different formulations were prepared as shown in Table 1: PPI control, PPI + starch (PS), PPI + fat (PF) and PPI + starch + fat (PSF). The preparation method was the same as given in Section 2.2. For chicken paste added samples, raw chicken pastes were blended with uncooked PSF paste in the proportions of 20 and 50% (Table 1).

Pastes were loaded on the rheometer plate. The temperature was set at 25 °C at the beginning of the test and heated until 95 °C at the rate of 4 °C/min. After holding for 30 s at 95 °C, samples were cooled down from 95 to 25 °C at the rate of 4 °C/min. At the end of the test, cooled samples were held for 30 s at 25 °C. Storage modulus (*G’*), Loss modulus (*G″*) and *tan δ* at were recorded. A little amount of mineral oil (Bio-Rad Laboratories, Rosedale, New Zealand) was applied to the sample edges to minimize the moisture loss.

#### 2.3.2. Frequency Sweeps

P control, PS, PF and PSF pastes were prepared as described in Section 2.2 and cooked in a boiling water for 10 min. PSF pastes with 20 and 50% raw chicken paste (20CHK and 50CHK) were prepared in the same manner as described in Section 2.2. Chicken paste was not cooked because denaturation would decrease the flow ability of samples. Viscoelastic parameters such as *G’*, *G″* and *tan δ* were determined at 25 ± 0.1 °C, with angular frequency increasing from 0.1 to 100 rad/s. Ten points were recorded within each decade.

#### 2.3.3. Shear Flow Behaviour Tests

Samples used in shear flow behaviour tests were the same as mentioned in the frequency sweep tests. Tests were carried out at 25 ± 0.1 °C, while the shear rate was ramped from 0.1 to 100 s^−1^. Shear-viscosity curves were obtained after testing, with 10 points shown within each decade.

### 2.4. Forward Extrusion Testing of PPI and Chicken Pastes

Forward extrusion tests were conducted with a textural analyser (TA.XT.plus, Stable Micro Systems, Godalming, UK) with a 50 kg load cell, using the method described by Kim et al. [29] and Zhu et al. [30] with minor modification. A device consisting of a syringe and piston was set up on the texture analyser (Supplementary Materials Figure S1). Four PPI-based pastes, 20CHK, 50CHK and chicken pastes were prepared as described in Section 2.2. They were carefully scooped to fill into a 60 mL polypropylene syringe with a spatula. The syringe was placed vertically on a heavy-duty platform (HDP) with a hole in the centre. The test was conducted by a single compression with a 61 mm cylindrical probe. Compression force-time curve was obtained, and the maximum compression force was defined as the extrusion hardness of each food paste.

The compression speed for the forward extrusion test was calculated based on a range of equations. The relationship between compression distance and paste length is shown in Equation (1).

(1) $$\frac{{\pi d}_{f}^{2}}{4}\times \mathrm{compression}\;\mathrm{distance}=\frac{{\pi d}_{n}^{2}}{4}\times \mathrm{paste}\;\mathrm{length}$$

where d_f_ is the diameter of the filament, which also equals to the syringe diameter in this study; d_n_ is diameter of the nozzle. Paste length refers to the length that paste extruded out from the syringe and nozzles.

If the extrusion time is controlled, Equation (1) can be modified to Equation (2), which shows the relationship between compression speed and extrusion speed. If extrusion speed is set, then the compression speed could be calculated by Equation (3).

(2) $$\frac{{\pi d}_{f}^{2}}{4}\times {\mathrm{Speed}}_{c}=\frac{{\pi d}_{n}^{2}}{4}\times {\mathrm{Speed}}_{e}$$

(3) $${\mathrm{Speed}}_{c}={\mathrm{Speed}}_{e}\times {\left(\frac{d_{n}}{d_{f}}\right)}^{2}$$

where Speed_c_ is the compression speed; Speed_e_ is the speed at which paste extruded out from the nozzle.

The diameter of syringe used in this study was 28.5 mm. The diameters of two selected nozzles were 1.54 and 2.16 mm. Thus, the compression speed was set as 0.04 mm/s for the 1.54 mm nozzle and 0.09 mm/s for the 2.16 mm nozzle. This referred to the extrusion speed of 15 mm/s for each nozzle size. The compression distance was 5 mm and the average compression force measured and calculated from triplicated observations.

### 2.5. 3D Printing Process

The printer used in this study was a newly assembled LVE 3D printer (Supplementary Materials Figure S2). It is a combination of a plastic filament 3D printer frame and an extruder unit. The frame of the printer belonged to Creality Ender-3 (Creality 3D, Shenzhen, China). However, the extruder unit was created based on the design from Pusch et al. [31].

In this study, all 3D models for experiments were downloaded from online sources, which are open to public access through 3D builder (Version 18.0.1931.0; Microsoft Co., Redmond, WA, USA). Then, they were loaded and sliced by Repetier Host (Version 2.1.4; Hot-World GmbH and Co. KG., Willich, Germany).

**Figure 1:**
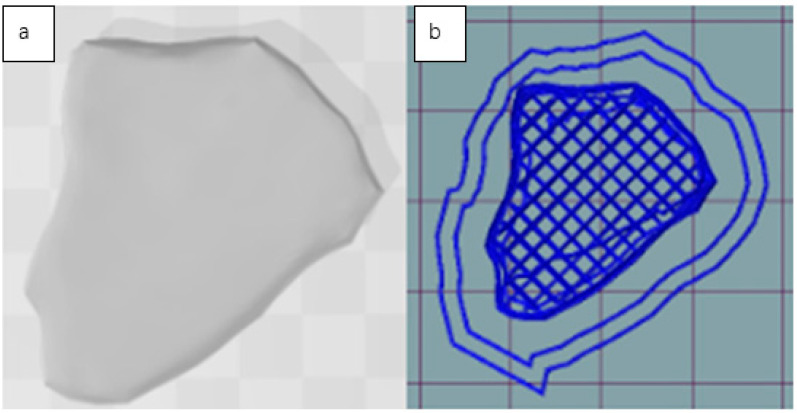
A 3D model of a chicken nugget shape downloaded through 3D builder (**a**) and sliced by Repetier Host (**b**).

PSF, 20CHK and 50CHK were selected for 3D printing based on their rheological properties. The pastes were prepared as described in Section 2.2 and filled into syringes. A small nugget shape sample (Figure 1) was printed using a large volume extrusion (LVE) 3D printer. The effect of nozzle size (1.54 and 2.16 mm) on the printability (ability to form layers) was observed along with the appearance of the printed samples. Printing was carried out at ambient temperature, with a printing speed of 15 mm/s and 100% infill density. Printed samples were cooked in sealed polypropylene bags in a boiling water bath. The structures of both raw and cooked samples were visually evaluated. Fibre formation was particularly noticed.

### 2.6. Statistical Analysis

The data presented in the results and discussions are the mean values of triplicated measurements. In forward extrusion tests, standard deviation (SD) is also presented. One-way analysis of variation (ANOVA) and Tukey’s pairwise comparisons were conducted by Minitab (version 18.1, Minitab Inc., State College, PA, USA) to analyse the significance of the data. Statistical significance was defined by a *p* value lower than 0.05.

## 3. Results and Discussion

### 3.1. Rheology

#### 3.1.1. Temperature Sweeps

##### PPI Pastes

**Figure 2:**
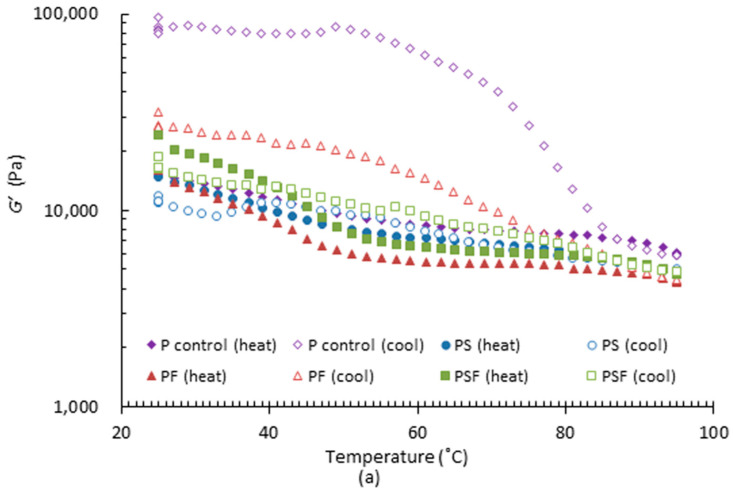

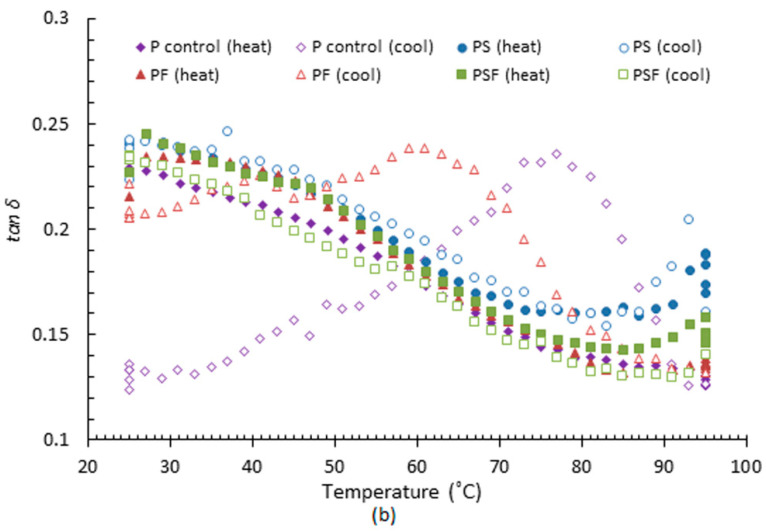
Viscoelastic properties of four PPI pastes during heating (25 to 95 °C) and cooling (95 to 25 °C) at rate of 4 °C/min. (**a**) Storage modulus (*G’*) and (**b**) *tan δ.* In the figure, P control represents PPI paste; PS represents PPI Paste containing starch; PF represents PPI paste containing fat; PSF represents PPI paste containing both starch and fat. Result shown was the mean value of triplicated tests.

In temperature sweep tests, the comparison of storage modulus (*G’*), loss modulus (data not presented) and loss factor (*tan δ*) of four PPI pastes are shown in Figure 2. Both *G’* and *G″* of all samples decreased during the heating process. They dropped down rapidly before 55 °C and then slowly decreased after that. A change in rheological properties in protein-based food is normally associated with protein denaturation. According to Shand et al. [32], the denaturation temperature of non-globulin and globulin fractions in lab-prepared PPI were 67 and 85 °C, respectively. However, there are no obvious changes on moduli at either temperature in Figure 2a. It was reported by Aryee et al. [33] that processing methods vary the characteristics of PPI products. The decreasing trend in moduli during heating is generally similar to the study by Moreno et al. [34], in which PPI with a greater denaturation degree showed a continuous reduction in *G’* and *G″* during heating. This was explained to be the result of the destruction of polar interactions, mainly leading to decreased moduli. Hence, the PPI used in this study is assumed to be already denatured to some extent due to processing. As demonstrated by Jiang et al. [35], denatured protein shows a higher extrudability since most native proteins have poor shape-holding capacity. This could be the reason that PPI paste can be manually extruded in the trials.

During cooling, *G’* and *G″* of the four pastes increased to different extents. A similar phenomenon was reported by Sun and Arntfield [36], who investigated the rheological properties of salt-extracted PPI at different temperatures. In their research, *G’* of all PPI samples increased steeply from 95 to 25 °C. The increasing curves of *G’* differ from this study. This could be caused by the different heating and cooling rates and sample compositions. As reported by Oyinloye and Yoon. [37], a slower cooling rate led to a faster increase in *G’* and *G″*. Moreover, different formulations influenced rheological properties. The storage and loss moduli for the samples with starch added (PS and PSF) returned to a slightly lower level than at the beginning of heating cycle. This finding suggested that starch limited the moduli change during heating and cooling. Therefore, cooling of PS and PSF can be considered as a roughly reverse process to heating. However, *G’* and *G″* of P control and PF were higher than the initial values before heating. As can be seen, viscoelastic moduli of P control and PF rose from approximately 90 to 50 °C, while *G″* of these two samples increased dramatically from 90 to 70 °C.

Phase changes during heating and cooling did not exist, since *tan δ* of all these samples was lower than 1 at all temperatures (Figure 2b). *Tan δ* of all these samples ranged between 0.1 and 0.25, which means samples were always predominantly elastic [38]. Before the heating process, the initial *tan δ* of all paste samples was between 0.2 and 0.25. During heating, all pastes showed, generally, a decreasing tendency in *tan δ*, indicating that the gel strength is reinforced. The reason was associated with the protein–protein interactions generated from temperature change, which contributed to a more elastic gel network [36]. *Tan δ* of P control and PF was lower than 0.15 at 95 °C. For PS and PSF however, *tan δ* rose slightly when the temperature was higher than 85 °C. During cooling, *tan δ* of PS increased dramatically at the beginning, dropping down afterwards and rising again when the temperature was below 83 °C. As for the PSF sample, *tan δ* steadily increased. The changes in *tan δ* of P control and PF during cooling were more complicated. *Tan δ* of PF increased drastically from 95 to 59 °C and decreased smoothly afterwards. PS, PF and PSF samples did not change hugely during the entire heating and cooling process. *Tan δ* of P control rose rapidly and dropped down after 77 °C to nearly 0.13, which is much lower than the initial *tan δ*. This indicates that the cooling procedure increased the gel firmness of the P control sample. In general, results show that starch added samples were suitable to be heated and cooled before printing. This is because they demonstrate a minor reduction in viscoelastic moduli and a similar *tan δ*, which potentially create a proper paste fluidity for printing [28]. More studies on plant protein-based food are needed to explore the interactions among protein, starch, and fat. A deeper understanding of component interactions helps to design formulations with ideal rheological properties for printing.

##### PPI-Chicken Paste

**Figure 3:**
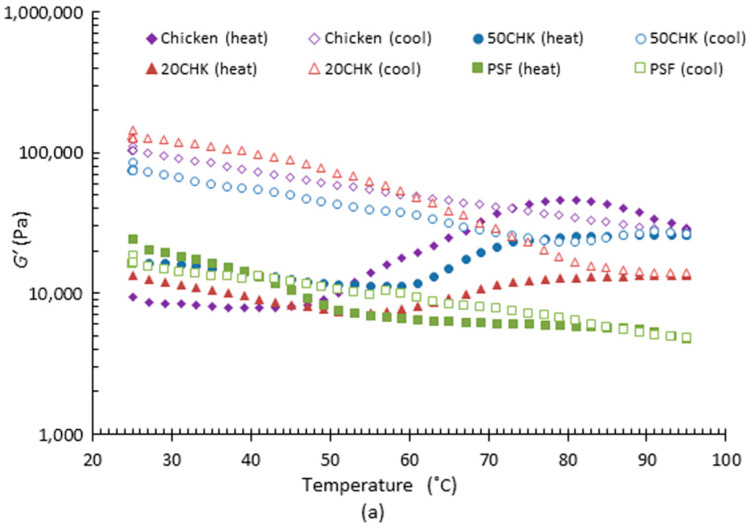

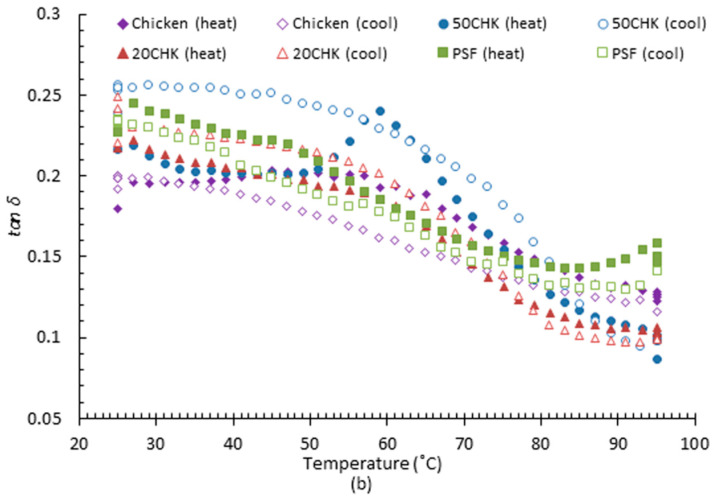
Viscoelastic properties of PPI and chicken mixture samples during heating (25 to 95 °C) and cooling (95 to 25 °C). (**a**) Storage modulus (*G’*) and (**b**) *tan δ*. In the figure, PSF represents PPI paste containing both starch and fat; 20CHK represents 20% chicken added into PSF paste; 50CHK represents 50% chicken added into PSF pastes. The result shown was the mean value of triplicated tests.

For chicken added samples, the change of viscoelasticity is shown in Figure 3. During both heating (50 °C onwards) and cooling, chicken and chicken paste added samples showed an increased G’ over that of PPI paste alone. *G’* of chicken and chicken added samples increased steeply when the temperature was between 60 and 80 °C (Figure 3a). A similar rheology investigation was reported by Rabeler and Feyissa [39], in which the *G’* of chicken breast showed a rapid increase from 60 to 80 °C. Minor differences in *G’* values reported in this study could be explained by the different protein contents of the meat samples. As reviewed by Lesiów and Xiong [40], the denaturation of majority of proteins, including myosin and myofibrils in chicken meat occurred between 35 and 40 °C. The denatured proteins started to aggregate and form gels with the continuously increasing temperature. Tornberg [41] indicated that denaturation by heating may have caused meat fibre contraction and connective tissue solubilization. Such changes formed a denser network that enhanced *G’* of meat during heating.

The variation of *G″* in the four samples showed a similar trend to *G’* in the entire process (data not shown). However, *G″* of chicken and 50CHK reduced slightly from 75 °C onwards during heating. *G″* of 20CHK had a slight increase during heating after 50 °C. The increase in *G’* and *G″* during heating declined with the increasing amount of chicken paste, indicating that meat protein has more sensitivity to temperature than PPI at a high temperature. Both *G’* and *G″* of chicken and chicken-added samples increased steeply after the heating and cooling process.

Compared with PSF and chicken, the extent of increase in *tan δ* after the entire process for the 20CHK and 50CHK samples were greater. This might demonstrate that interactions between plant and animal proteins simulate the change in *tan δ* during temperature change. Rheological properties of emulsion gel systems containing pea protein and animal protein have been studied by Graca, Raymundo and de Sousa [42]. Their investigation showed that *tan δ* of a food sample containing pea protein and collagen protein in a ratio of 50:50 increased to over 1 from 40 °C during heating. It represented a breakdown of the original emulsion structure. The deformation was maintained until 80 °C when a new gel structure formed (*tan δ* < 1). This trend did not appear in samples containing either a higher amount of pea protein or purely collagen protein. It could explain why only 50CHK exhibits an increasing *tan δ* between 50 and 60 °C in this study (Figure 3b).

#### 3.1.2. Frequency Sweeps

**Figure 4:**
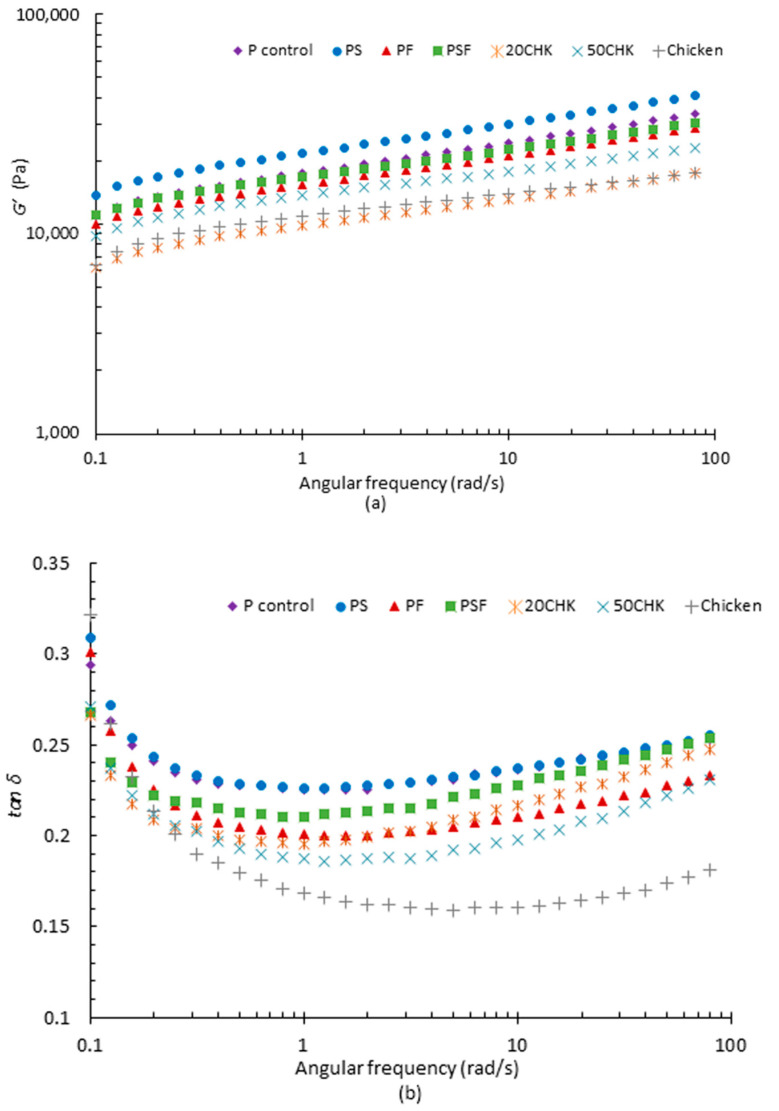
Viscoelastic properties of different PPI pastes (**a**) Storage modulus (*G’*) and (**b**) *tan δ*. In the figure, P control represents PPI paste; PS represents PPI Paste containing starch; PF represents PPI paste containing fat; PSF represents PPI paste containing both starch and fat; 20CHK represents 20% chicken added into PSF paste; 50CHK represents 50% chicken added into PSF paste. Result shown was the mean value of triplicated tests.

During frequency sweeps, *G’* and *G″* of four PPI pastes both progressively increased with the growing angular frequency (Figure 4). In addition, *G’* was always higher than *G″*, indicating that these pastes show a weak gel behaviour [43]. For both *G’* and *G″*, PS paste showed the highest value than other pastes during the whole frequency sweep test among all plant protein only samples (Figure 4a; data for *G″* not presented). Interestingly, cooked P control and PF pastes did not exhibit a high value for viscoelastic moduli after the heating and cooling cycle, similar to the temperature sweeps (Figure 2). This may be because cooked samples were cooled at a different rate than in the conditions of the temperature sweep. Higher *G’* and *G″* values could be caused by adding starch, which potentially decrease the fluidity [28,44]. In contrast, the addition of fat could enhance fluidity, which was agreed by Lille et al. [45]. This phenomenon may benefit the printing process.

Similar to PPI pastes, *G’* of 20CHK, 50CHK and chicken was higher than *G″*, and both increased progressively during the frequency sweep. It shows that chicken added samples also behave similar to a weak gel. *G’* and *G″* of cooked PSF pastes were reduced by the addition of raw chicken paste, with 20% chicken paste showing lower values than 50% during the experiment. Raw chicken showed a higher *G’* than 20CHK, but a lower value than 50CHK (Figure 4a). *G″* of 20CHK was lower than chicken when the angular frequency was lower than 0.6 rad/s but surpassed it afterwards (Figure 4b). This demonstrates that the 20CHK sample had the highest fluidity among all three samples, which demonstrates a better flowability [28].

All seven pastes showed a decreasing tendency on *tan δ* below 1 rad/s (Figure 4b). In this stage, *tan δ* of chicken paste showed the sharpest reduction. When the angular frequency is over 1 rad/s, *tan δ* of PPI and chicken added pastes increased slowly. For chicken paste, the *tan δ* value remained stable between 1 and 10 rad/s and rose slightly to a higher angular frequency. Savadkoohi et al. [46] explained that this change was associated with the damage to the gel network. In their research, similar plateaus of *tan δ* of chicken-based samples in a higher angular frequency range (1 to 100 rad/s) was observed. PF expressed the lowest *tan δ* among all PPI pastes after the frequency sweep. *Tan δ* of PSF showed minimal change before and after the whole process.

#### 3.1.3. Shear Flow Behaviour

**Figure 5:**
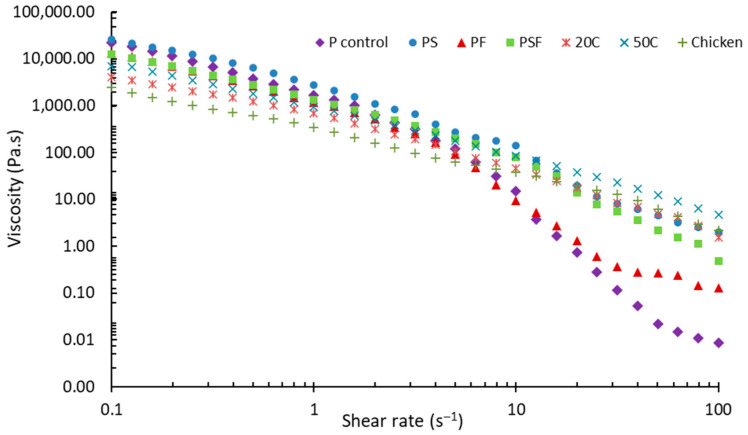
Apparent viscosity of samples at shear rate between 0.1 and 100 s^−1^. Comparison of shear viscosity curve of PPI pastes, chicken and PPI-chicken pastes. In the figure, P control represents PPI paste; PS represents PPI paste containing starch; PF represents PPI paste containing fat; PSF represents PPI paste containing both starch and fat; 20CHK represents 20% chicken added into PSF paste; 50CHK represents 50% chicken added into PSF paste. The result shown was the mean value of the triplicated tests.

In this study, all samples were pseudoplastic materials, showing a shear-thinning behaviour (Figure 5). This indicates that all samples are suitable to extrude, which is in agreement with the findings of Lipton [47]. The reduced viscosity caused by applied shear force enables food gels to be extruded from nozzles [35]. Among all PPI pastes, PS showed higher viscosity than the three other pastes (Figure 5). This indicates that starch increases the viscosity, which could be explained on the basis that cooked starch absorbs water and forms an intensive gel structure [48,49]. However, adding both starch and fat did not negatively influence the flow behaviour. This might be because the addition of fat reduced the viscosity, as PSF and PF samples both showed low initial viscosity among all the PPI pastes. A similar finding was reported by Lille et al. [45], namely that a food formulation with semi-skimmed milk powder was less viscous than with skimmed milk powder. In addition, the viscosity of the paste was decreased when the raw chicken paste was added.

At a shear rate higher than 10 s^−1^, PPI pastes (except the PS sample) become less viscous than chicken-added pastes and chicken. The most drastic reduction existed in the shear-viscosity curve of the P control paste, showing that P control paste is easy to deform when a shear force is added. A slight infinite shear viscosity plateau was shown in PF when the shear rate was above 40 s^−1^ (Figure 5).

As phase change was not involved during rheological testing in this study, shear-flow behaviour can be an important parameter to evaluate the sample’s extrudability. According to Hölzl et al. [50], the viscosity of bio-ink for extrusion-based printing can range from 3 × 10^−2^ to 6 × 10^4^ Pa s. In addition, a high zero-shear viscosity was also considered as a suitable property for extrusion type printing [51]. Nevertheless, Wang et al. [28] claimed that food material showed a poor printing performance if the zero-shear viscosity was too high. In their research, a food gel with zero-shear viscosity (viscosity at shear rate 0.1 s^−1^) around 30,000 Pa s was extrudable but not capable of expressing a proper printing appearance. In this study, the zero-shear viscosity of PS and P control pastes are both close to 30,000 Pa s (25,723 and 22,427 Pa s, respectively), which potentially means these two pastes are not suitable for a smooth 3D printing process.

### 3.2. Forward Extrusion Test

The extrusion force of various samples was measured by a forward extrusion test. As shown in Table 2, paste samples (except PS) showed a lower extrusion force from a 2.16 mm nozzle than a 1.54 mm nozzle. It was also shown by Zhu et al. [30] that tomato puree exhibited lower extrusion stress through a bigger (1.2 mm) nozzle than a smaller (0.8 mm) one. These findings suggest that a bigger nozzle is easier for extrusion. Raw chicken paste was not able to be extruded from a 1.54 mm nozzle. The reason for this is that the presence of big particles constrains the flow, which leads to big variations in different tests. It suggests that mincing at 556× *g* is not enough to break down some big muscle particles in chicken paste. Mincing at a higher rotation rate or filtering out big muscle particles may be helpful to produce smooth flows. However, it would possibly lead to a low shape-forming capacity during extrusion (as preliminary trials). Hence, chicken paste was not suitable to be directly used for 3D printing in this study. However, it can be added into PPI based pastes since 20CHK and 50CHK samples were able to extrude from both nozzle sizes. The P control paste exhibited the lowest extrusion force, demonstrating that it was easiest to extrude. The reason might be that the viscosity of the P control paste decreased dramatically at a high shear rate (Figure 5). PF showed the highest extrusion force of all the samples (Table 2).

A correlation between extrusion force (or extrusion stress) and rheological properties was shown by Zhu et al. [30]. They pointed out that the extrusion stress of food samples expressed a linear correlation with flow stress. A higher flow stress contributes to higher extrusion stress. However, there was no correlation between extrusion stress and viscoelastic properties. The reason was assumed to be that viscoelastic properties refer to the characteristics of a sample in a non-deforming stage, while flow stress and extrusion stress are both parameters related to deformation. Such correlation was suitable for various water-based food pastes, but not for oil-based food pastes. In addition, extrusion force was related to the materials’ printability, which is defined as the capacity of deposited materials to support their own weight [21]. Kim et al. [29] showed that the printability of hydrocolloid samples was positively correlated with the extrusion force. According to their finding, hydrocolloids with a higher methylcellulose concentration exhibited an increasing extrusion force, which simultaneously led to a lower deformation rate of a printed cylinder shape. Nevertheless, this correlation was not suitable for food samples with multiple ingredients. This is because interactions among ingredients creates a complex food matrix, which deserves further investigations. Currently, there is no available research that systematically assesses the relationship between extrusion force and printing performance. It is still necessary to correlate extrusion force and printing experiments, especially for food with numerous ingredients and complex structures.

### 3.3. Printing Performance

#### 3.3.1. Appearance of Printed Meat Analogues in Different Formulations and Nozzle Sizes

**Figure 6:**
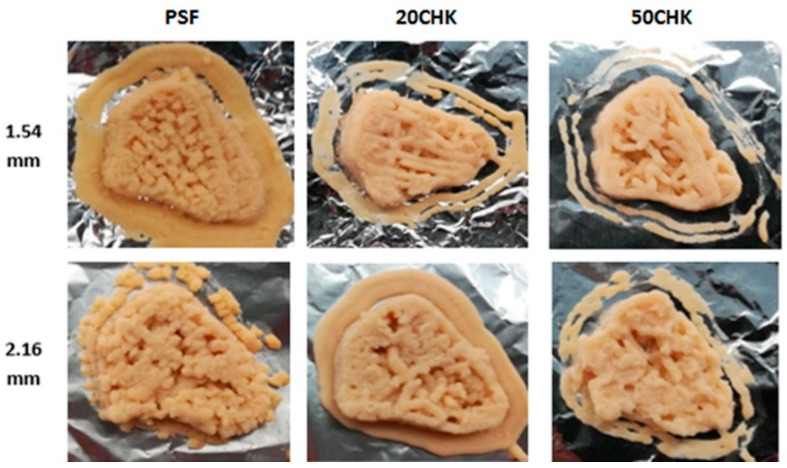
Printed meat analogues using 1.54 and 2.16 mm nozzles (15 mm/s, 100% infill). PSF represents PPI paste containing both starch and fat; 20CHK represents 20% chicken added into PSF paste; 50CHK represents 50% chicken added into PSF paste.

Printed samples with a small chicken nugget shape are shown in Figure 6. In general, both PSF and chicken paste added samples formed more desirable shapes through a 1.54 mm nozzle, compared with a 2.16 mm. Since PSF, 20CHK and 50CHK all showed lower extrusion force with a 2.16 mm nozzle size, it may indicate that a lower extrusion force results in lower printability. This agrees with the finding of Kim et al. [29]. During printing through a 2.16 mm nozzle, the poor printing performance could be related to the shear rate and viscosity. The relationship between shear rate and viscosity in extrusion printing can be shown by Equations (4)–(6) [52]:

(4) $$\dot{\gamma }=\frac{4Q}{{\pi r}^{3}}$$

(5) $$Q={\pi r}^{2}\times {\mathrm{Speed}}_{e}$$

(6) $$\dot{\gamma }=\frac{4\times {\mathrm{Speed}}_{e}}{r}=\frac{8\times {\mathrm{Speed}}_{e}}{d}$$

where $\dot{\gamma }$ is shear rate; Q is the volumetric flow rate, referred to extrusion rate in this study; r is the radius of the nozzle; Speed_e_ is the extrusion speed; d is the diameter of the nozzle.

Therefore, increasing the nozzle diameter with a controlled printing speed leads to a lower shear rate, resulting in a higher sample viscosity (Figure 5). It is assumed that higher viscosity causes poor extrusion behaviour. Although there is no available research that quantifies the relationship between shear viscosity and printability, similar investigations were reported by Wang et al. [28] showing that fish surimi with a high viscosity would reduce the extrusion smoothness. In this study, the viscous extrusion flow from a 2.16 mm nozzle contributed to an inconsistent deposition line and exhibited a less desirable appearance. Similar findings were reported by Yang et al. [53]. They reported that a bigger nozzle tended to result in poorer printing quality. Wang et al. [28] also tested printing performances through different nozzle diameters. In contrast to this study, however, they found that a printed sample from a smaller nozzle demonstrated poorer printing performance than from a bigger nozzle. The reason might be that printing through a small nozzle demanded a higher pressure, which caused an extremely low viscosity and a low shape-building capacity.

The printing smoothness declined with the increasing amount of chicken paste. Printed 50CHK samples through both nozzle sizes show many printing defects. This is associated with the hypothesis that a difference in smoothness of PSF paste and chicken paste may lead to a heterogeneous mixture and non-continuous flow during extrusion from nozzles. For meat paste samples, Dick et al. [54] recommended the use of nozzle sizes bigger than 2 mm to enable extrusion of some components containing big particles. A similar finding was shown in Section 3.2, namely that big particles in chicken blocked the 1.54 mm nozzle and stopped the extrusion process. Although 50CHK and 20CHK showed extrudability through a 1.54 mm nozzle, varied flow resistance of a chicken portion and a plant protein portion could cause a non-smooth extrusion. PSF paste was able to deposit bottom layers stably. Although printing defects also appeared on the surface layer, the general nugget shape based on the 3D model was formed. The reason could be that PSF paste has a more uniform structure than 50CHK and 20CHK samples.

#### 3.3.2. Appearance and Macrostructure of Printed Meat Analogues after Cooking

**Figure 7:**
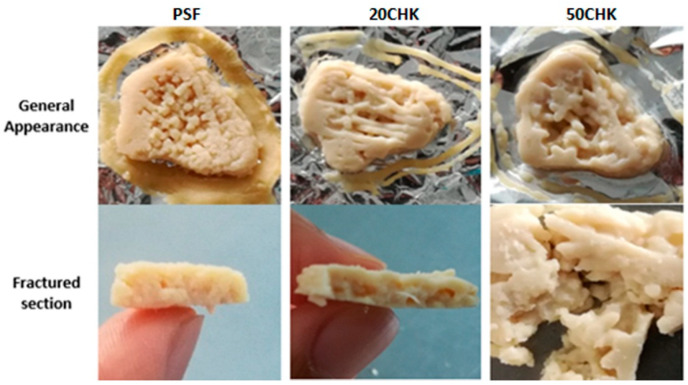
Printed meat analogues using 1.54 mm nozzle after cooking. PSF represents PPI paste containing both starch and fat; 20CHK represents 20% chicken added into PSF paste; 50CHK represents 50% chicken added into PSF paste. Printed samples were cooked in boiling water for 10 min.

The comparison of three cooked samples is presented in Figure 7. Chicken paste added samples showed a more acceptable colour after boiling in water. The shapes of the printed samples were slightly damaged after cooking. Although heat-sealed bags were used to prevent damage of the shape, the edges of the printed samples were not protected perfectly. Lipton et al. [55] tried cooking printed meat in a controlled vapour oven. The overall shape of printed turkey meat was protected from being damaged by the package. Nonetheless, cooking in a controlled vapour oven caused inward shrinkage of the meat, which made the shape bow upwards. To improve the printability of meat analogues, shape-maintenance after cooking is a challenge that needs to be overcome.

Fibrous structure formation is a pivotal characteristic to assess the quality of meat analogue. As found from fractured sections, PSF sample was insufficient to form a fibrous structure without adding chicken (Figure 7). According to previous studies, the fibrous structure of PPI was formed in the thermal extrusion system above 120 °C [25]. In a shearing processing system, the same conditions of temperature were required [56]. To develop a printed product from a PPI based formulation without adding meat, the post-printing cooking method should be changed. This is because the temperature of the boiling water bath (100 °C) was inadequate to build fibrous structures. Pan-frying, microwaving and baking were suggested, since these methods enable cooking samples at a higher temperature. Moreover, the addition of some ingredients such as wheat gluten would help fibre formation [56]. Fibres were found in 20CHK and 50CHK, indicating that meat fibres were generally provided by chicken paste. More fibres were found in 20CHK than the 50CHK sample because of its stable printed shape. This is associated with the proper extrusion and deposition performance of the 20CHK sample. Thus, 20CHK paste is considered as the optimal material for printing in this study.

## 4. Conclusions

This study investigates the printing of 3D nugget shapes by using plant-only and plant-meat-based formulations. PPI combined with maize starch, beef fat and water expressed suitable rheological properties for extrusion-based 3D printing. Both PPI paste and PPI chicken paste showed a weak gel behaviour according to rheology tests. The addition of maize starch increased the viscosity of food paste, while it minimized the moduli change during temperature sweep. The combination of chicken and PPI-based paste reduced the viscosity change with the increasing shear rate. The addition of raw chicken paste to cooked PPI–based paste was recommended, since it provided a suitable flow behaviour. Forward extrusion tests helped understand the general extrusion difficulties of different samples. However, the correlation between the extrusion force and other characteristics was not identified in this study.

Printing through a 1.54 mm nozzle showed better 3D shape forming capacity than a 2.16 mm nozzle. It was explained that a bigger nozzle size led to a higher shear viscosity, resulting in poor printability. The PPI-paste sample without chicken showed a more desirable appearance, while the addition of chicken paste into PPI paste created a fibre-like structure. Considering both printing performance and fibre formation, PPI paste with 20% chicken was selected as an optimal formulation.

The printing speed in this study was set as 15 mm/s, which is slower than many non-food materials for 3D printing. To scale up and reach the industrial requirements, it is necessary to develop the methods that allow printing of plant protein-based materials at a high speed.

## Figures and Tables

**Table 1:** Pea protein isolate (PPI) based paste and PPI-chicken paste (PCP) formulations.

Ingredients	Percentage (% *w*/*w*, Wet Basis)					
	P Control ^1^	PS ^1^	PF ^1^	PSF ^1^	20CHK ^1^	50CHK ^1^
PPI	30	26.4	27.6	24	19.2	12
Starch	0	3.6	0	3.6	3.6	3.6
Fat	0	0	2.4	2.4	2.4	2.4
Chicken paste	0	0	0	0	19.2	48
Soy lecithin	1	1	1	1	1	1
Water	69	69	69	69	54.6 ^2^	33 ^2^

^1^ P control represents pea protein isolate paste, PS represents PPI paste with starch, PF represents PPI paste with fat, PSF represents PPI paste with both starch and fat. 20CHK represents 20% chicken added into PSF paste; 50CHK represents 50% chicken added into PSF pastes. ^2^ The amount of chicken paste and water are based on moisture content analysis of the raw chicken pastes. The moisture of the chicken paste was 75.30 ± 0.23% measured by hot air oven method. The total moisture of these PCP samples was kept as 69%, which is similar to PPI pastes.

**Table 2:** The extrusion force of tested materials with 1.54 and 2.16 mm nozzle sizes.

Samples ^3^	Extrusion Force (N) ^1,2^	
	Nozzle Size	
	1.54 mm	2.16 mm
P control	57.74 ± 1.86 ^a^	49.91 ± 1.98 ^a^
PS	73.47 ± 4.30 ^b^	83.64 ± 2.18 ^d^
PF	141.10 ± 9.43 ^e^	98.80 ± 2.13 ^e^
PSF	87.88 ± 3.63 ^c^	62.13 ± 2.85 ^b^
20CHK	106.31 ± 3.06 ^d^	83.22 ± 2.50 ^d^
50CHK	84.27 ± 0.85 ^b,c^	74.07 ± 0.54 ^c^
Chicken	N/A	67.73 ± 1.78 ^b^

^1^ Results are shown as means ± SD (*n* = 3). ^2^ According to Tukey’s pairwise comparison, different letters in each column show a significant difference (*p* < 0.05). ^3^ P control represents PPI paste; PS represents PPI Paste containing starch; PF represents PPI paste containing fat; PSF represents PPI paste containing both starch and fat; 20CHK represents 20% chicken added into PSF paste; 50CHK represents 50% chicken added into PSF past.

## Data Availability

We exclude this statement.

## Supplementary Materials

The following are available online at www.mdpi.com/article/10.3390/foods11030478/s1, Figure S1. Design of forward extrusion test: A syringe and piston device attached to a texture analyser. (1) The framework of textural analyser (TA.XT.plus, Stable Micro Systems, UK). (2) A 61 mm cylindrical probe. (3) A piston. (4) A syringe. (5) HDP/90 platform with a hole in the centre. (6) A container to collect the paste extruded out from syringe.; Figure S2. Photograph of LVE 3D printer used in this study. (1) Framework of Ender-3 3D printer. (2) Extruder unit. (3) Gears. (4) Motor. (5) Nozzle holder. (6) Platform. (7) Conveyor belts, controlling the movement to x, y, z axis directions. (8) Operation menu. (9) USB connection to computer.
