# Supplementary material for: 3D Printing of Textured Soft Hybrid Meat Analogues
Source: Foods. 2022 Feb 6;11(3):478. doi: 10.3390/foods11030478 (PMC8834039; doi:10.3390/foods11030478)
Supplement: Supplementary file 1 [file foods-11-00478-s001.zip › foods-1505853-supplementary.pdf]

Supplementary data

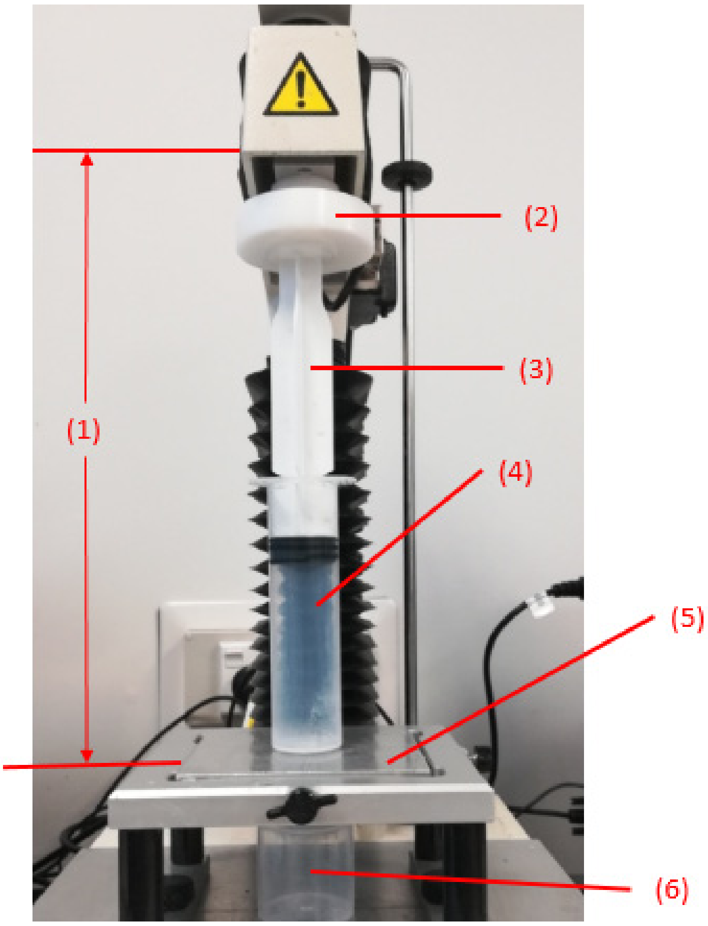

Figure S1. Design of forward extrusion test: A syringe and piston device attached to a texture analyser.

(1) The framework of textural analyser (TA.XT.plus, Stable Micro Systems, UK). (2) A 61 mm cylindrical probe. (3) A piston. (4) A syringe. (5) HDP/90 platform with a hole in the centre. (6) A container to collect the paste extruded out from syringe.

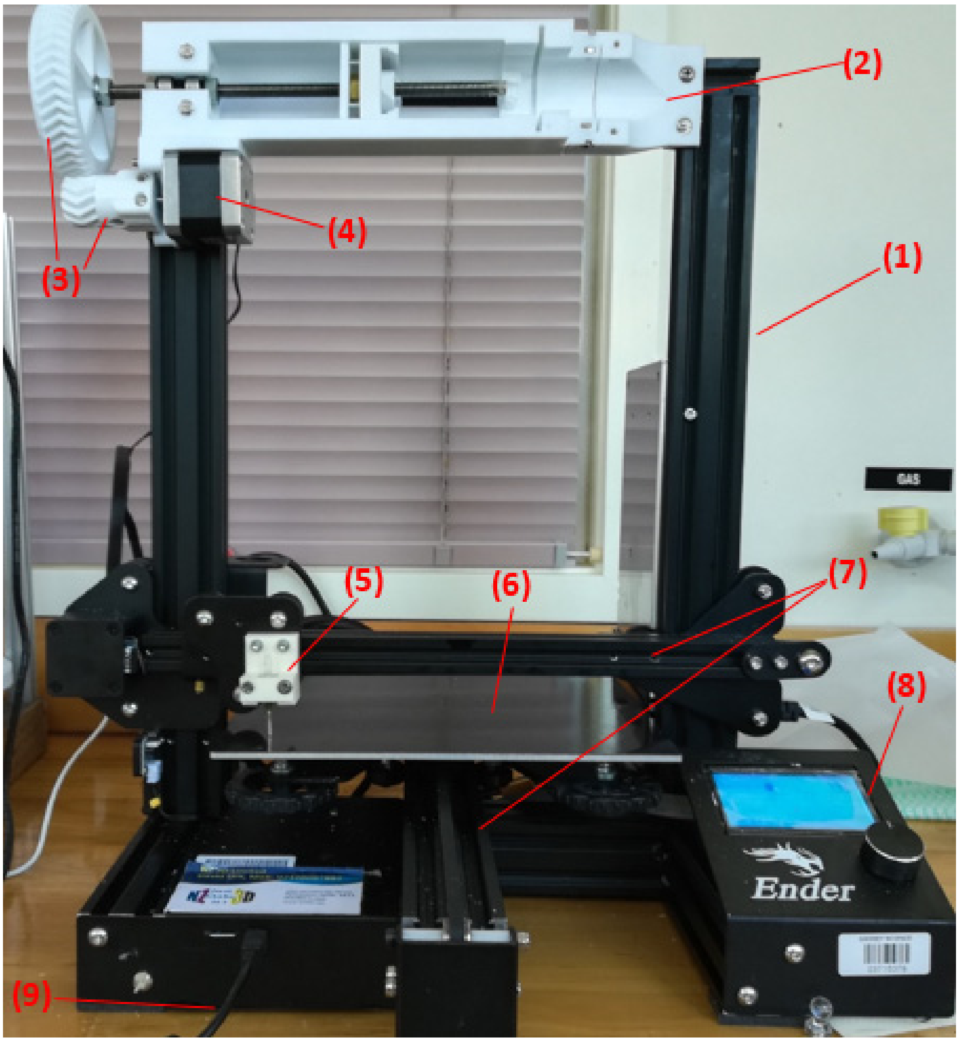

Figure S2. Photograph of LVE 3D printer used in this study.

(1) Framework of Ender-3 3D printer. (2) Extruder unit. (3) Gears. (4) Motor. (5) Nozzle holder. (6) Platform. (7) Conveyor belts, controlling the movement to x, y, z axis directions. (8) Operation menu. (9) USB connection to computer.
